# Systems biology and metabolic engineering of *Rhodococcus* for bioconversion and biosynthesis processes

**DOI:** 10.1007/s12223-021-00892-y

**Published:** 2021-07-03

**Authors:** Eva Donini, Andrea Firrincieli, Martina Cappelletti

**Affiliations:** Department of Pharmacy and Biotechnology, University of Bologna, Via Irnerio 42, 40126 Bologna, Italy

## Abstract

*Rhodococcus* spp. strains are widespread in diverse natural and anthropized environments thanks to their high metabolic versatility, biodegradation activities, and unique adaptation capacities to several stress conditions such as the presence of toxic compounds and environmental fluctuations. Additionally, the capability of *Rhodococcus* spp. strains to produce high value-added products has received considerable attention, mostly in relation to lipid accumulation. In relation with this, several works carried out *omic* studies and genome comparative analyses to investigate the genetic and genomic basis of these anabolic capacities, frequently in association with the bioconversion of renewable resources and low-cost substrates into triacylglycerols. This review is focused on these *omic* analyses and the genetic and metabolic approaches used to improve the biosynthetic and bioconversion performance of *Rhodococcus*. In particular, this review summarizes the works that applied heterologous expression of specific genes and adaptive laboratory evolution approaches to manipulate anabolic performance. Furthermore, recent molecular toolkits for targeted genome editing as well as genome-based metabolic models are described here as novel and promising strategies for genome-scaled rational design of *Rhodococcus* cells for efficient biosynthetic processes application.

## Introduction

*Rhodococcus* genus comprises Gram-positive, non-motile, non-sporulating, aerobic bacteria, with a high G + C content and a mycolic acid-containing cell wall (Martínková et al. 2008). Members of *Rhodoccocus* genus are widely distributed in soil, water, and marine sediments, due to their metabolic flexibility and their tolerance to various stresses (e.g., presence of toxic metals and metalloids, desiccation, low nutrient availability, high concentration of organic pollutants). Only a few strains are pathogens of plants and animals (including humans) and belong to *Rhodococcus fascians* and *Rhodococcus equi* species, respectively (Cappelletti et al. 2019). The outstanding metabolic diversity and strong persistence of this genus is associated to peculiar features of their cell surface (i.e., hydrophobicity, mycolic acids content, and fatty acid composition rearrangement) and, in some cases, to the presence of large and complex genomes containing a multiplicity of genes involved in unique catabolic and anabolic processes (De Carvalho et al. 2014; Laczi et al. 2015; Orro et al. 2015; Cappelletti et al. 2016; Kis et al. 2017; Presentato et al. 2016, 2018a, b, c, 2020). In relation to this, *Rhodococcus* spp. strains are capable of performing biotransformation and biodegradation of many organic and xenobiotic compounds, such as hydrocarbons and chlorinated hydrocarbons, naphthenic acids, nitroaromatics, and pharmaceuticals (e.g., diclofenac and sulfamethoxazole) (Auffret et al. 2009; Cappelletti et al. 2010, 2015, 2016, 2018; Orro et al. 2015; Presentato et al. 2018a, b, c; Weidhass et al. 2009; Ivshina et al. 2019; Tyumina et al. 2019; Larcher and Yargeau 2011). Due to these wide metabolic capabilities and stress resistance/tolerance, *Rhodococcus* strains are considered ideal candidates for biotechnological applications in environmental remediation and pharmaceutical and chemical industries (Bell et al. 1998; Busch et al. 2019; Ceniceros et al. 2017; Kis et al. 2015; Larkin et al. 2005; van der Geize and Dijkhuizen 2004, Patek et al. 2021). In particular, extensive research focused on the production and accumulation of neutral lipids, mostly triacylglycerols (TAGs), in *Rhodococcus* through the bioconversion of industrial wastes and lignin biomass feedstocks. TAGs are energy-rich compounds formed by a molecule of glycerol esterified with three fatty acids chains. They can be used for biofuel generation after bacterial cell extraction through conventional chemical and physical extraction methods (e.g., sonication, solvent extraction) or through biological approaches (e.g., enzymatic cell lysis, phage-based extraction) (Hwangbo and Chu 2020).

Other valuable compounds that can be produced by *Rhodococcus* spp. strains are glycolipid biosurfactants, carotenoids, polyhydroxyalkanoates (PHAs), metal-based nanomaterials, and novel antimicrobials (Cappelletti et al. 2020). While experimental works have mostly investigated culture conditions and suitable substrates for efficient biotechnological applications, the identification of specific genes/proteins involved in these biosynthetic pathways has been for a long time hindered by the lack of efficient molecular methods/tools generally applicable to *Rhodococcus* spp. strains (Cappelletti et al. 2010). Transformation protocols in *Rhodococcus* spp. strains initially relied on protoplast-mediated procedures (Singer and Finnerty 1988; Duran 1998; Desomer et al. 1990). More recently, conjugation and electroporation methods have been successfully applied, although the results were shown to greatly vary within the *Rhodococcus* genus at species and even strain level (Shao et al. 1995; Sekizaki et al. 1998; Kalscheuer et al. 1999).

The decreasing cost of sequencing technologies and increasing number of complete genome sequences of different *Rhodococcus* spp. strains available in the databases allowed the in silico detection of numerous genes/enzymes involved in biosynthetic processes mostly leading to the accumulation of lipids. In this regard, genes predicted to be involved in the synthesis and accumulation of triacylglycerols, wax esters, polyhydroxyalkanoates (PHAs), and fatty acids were described in *R. jostii* RHA1, *R. opacus* PD630, *R. opacus* B4, *R. erythropolis* PR4, *R. equi* 103S, and *R. fascians* F7 (Hernandez et al. 2008; Alvarez et al. 2013; Cappelletti et al. 2019).

Among the different *Rhodococcus* strains that have been described in the literature, *R. opacus* PD630 and *R. jostii* RHA1 have been the most extensively studied for their biosynthetic and bioconversion activities due to the following reasons, (i) the peculiar capacity of using lignocellulose-based sugars along with toxic lignin-derived aromatic compounds; (ii) the high content of valuable lipids, mostly triacylglycerols (TAGs), that can accumulate from different carbon sources; (iii) the rapid growth rate; and finally, (iv) a good genetic tractability (DeLorenzo et al. 2017). Indeed, under nitrogen-limited conditions (i.e., reduced amount of nitrogen source added to the growth medium) and optimized fed-batch fermentation conditions, an accumulation of TAGs corresponding to around 75% of the cell dry mass can be achieved in *R. opacus* PD630 cells growing on glucose (Kim et al. 2019). On the other hand, some genetic manipulation strategies were developed and successfully applied to *R. opacus* PD630 and *R. jostii* RHA1 that allowed both genetic analyses and strain performance improvement.

**Fig. 1:**
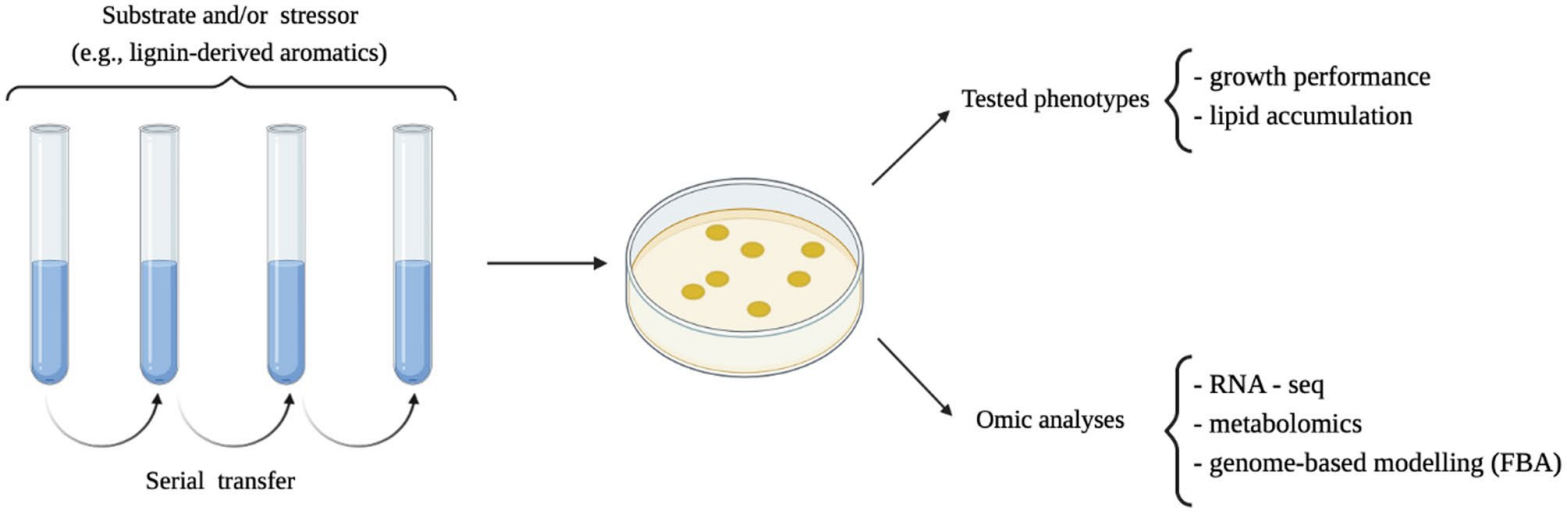
Schematic representation of the adaptive laboratory evolution (ALE) method applied to *Rhodococcus* spp. strains and types of analyses performed on the evolved strains. Figure created with BioRender.com

**Fig. 2:**
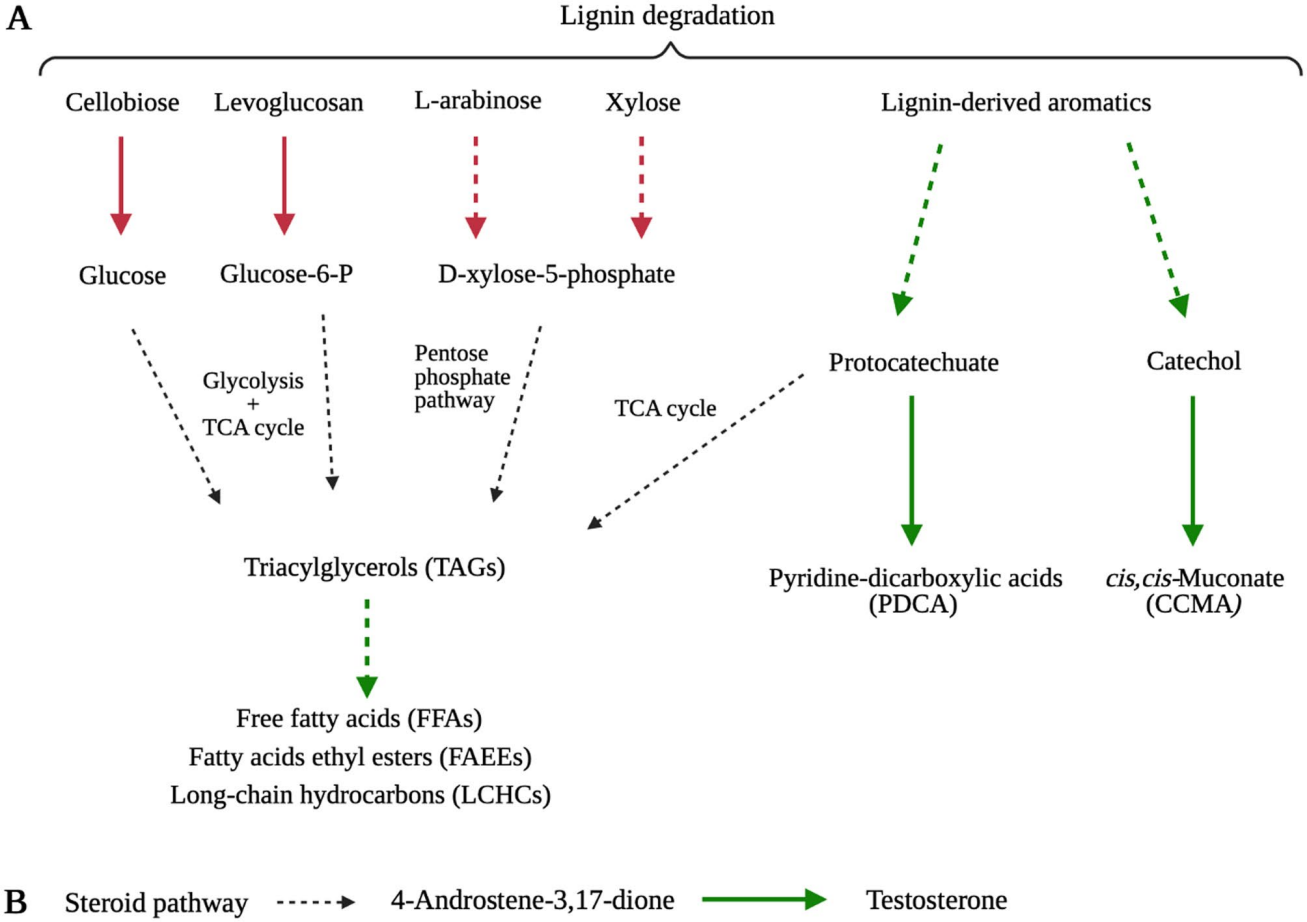
Main pathways in *Rhodococcus* spp. strains leading to the production and accumulation of valuable compounds from lignin degradation products (**A**) and steroid (**B**). The colors of the arrows indicate the methodological approaches used to optimize/improve the specific biosynthetic capability, i.e., red and green arrows correspond to heterologous expression and genome-based metabolic engineering, respectively. Dashed arrows represent several reactions, while solid arrows indicate a single reaction

Previous reviews summarize the regulation and the metabolic pathways involved in lipid accumulation in *Rhodococcus* (Alvarez et al. 2019), the different valuable compounds biosynthesized by members of this genus (Cappelletti et al. 2020) and those specifically derived from lignocellulose/lignin bioconversion (Anthony et al. 2019). Conversely, the present review is focused on the main genetic manipulation and metabolic engineering strategies applied to modify this genus strains to extend and optimize their biosynthetic capabilities and biotechnological applications (summarized in Table 1; Figs. 1 and 2). Some indications on novel genome editing tools (i.e., based on CRISPR/Cas9 and recombineering systems) are also reported as new strategies for rational genetic engineering of *Rhodococcus* spp. strains for biosynthetic purposes.

**Table 1:** List of *Rhodococcus* strains engineered to expand the substrate utilization range and to improve the biosynthesis of valuable compounds

Strain	Substrate	Final product	Experimental approach	Additional details on experiments and molecular methods	Reference
*R. opacus* MITXM-61 (expressing *xylA* and *xylB* from *Streptomyces padanus*)	Glycerol	TAGs	Adaptive laboratory evolution (ALE)	Sequential transfers in flask cultivations supplemented with 100 g/L of glycerol to improve glycerol utilization; variable N concentrations	Kurosawa et al. (2015b)
*R. opacus* MITXM-61 (expressing *xylA* and *xylB* from *Streptomyces padanus*)	Lignocellulose and analogs/derivatives	TAGs^a^	Adaptive laboratory evolution (ALE)	Three consecutive ALE passages, each one consisted of four transfer steps in the presence of increasing concentrations of lignin, 4-HB or syringaldehyde	Kurosawa et al. (2015a)
*R. opacus* PD630	Phenol	TAGs^a^	Adaptive laboratory evolution (ALE)	PD630 cells were grown for forty successive subcultures with increasing concentrations of phenol as sole carbon source; N-limited conditions	Yoneda et al. (2016)
*R. opacus* PD630	Lignin-derived aromatics	TAGs^a^	Adaptive laboratory evolution (ALE)	Serial passages in the presence of combinations of protocatechuate, guaiacol, phenol, 4-hydroxybenzoate, and vanillate as carbon sources to develop strains with optimized utilization of lignocellulose-derived aromatics; N-limited conditions	Henson et al. (2018)
*R. opacus* PD630	Cellobiose	TAGs	Heterologous expression of *bglABC* operon from *Thermobifida fusca*	Plasmid pEC-K18*mob2::bglABC*_TF_ (Km^R^ and inducible promoter *P*_*lac*_)	Hetzler and Steinbüchel (2013)
*R. opacus* PD630 *R. jostii* RHA1	Xylose	TAGs	Heterologous expression of the genes *xylA* and *xylB* from *Streptomyces lividans* TK23	Plasmid pXYLAB (Km^R^; pTACHis18 containing *xylA* and *xylB* under the inducible promoter *P*_*tac*_); N-limited conditions	Xiong et al. (2012)
*R. opacus* PD630	Xylose	TAGs	Heterologous expression of the genes *xylA* and *xylB* from *Streptomyces padanus* MITKK-103	Plasmids pAL358 (Gm^R^) and pAL307 (Spec^R^) used as cloning vectors for the *S. padanus* genomic library preparation	Kurosawa et al. (2013)
*R. opacus* PD630	l-arabinose	TAGs	Heterologous expression of *araBAD* operon from *Streptomyces cattleya* NRRL 8057	Plasmid pASC8057 harboring *araB, araD*, *araA*, derived from *Rhodococcus/E. coli* shuttle vector pX0 (Spec^R^)	Kurosawa et al. (2015c)
*R. jostii* RHA1	l-arabinose	TAGs	Heterologous expression of *araBAD* and *araFGH* operons from *Escherichia coli* K12 MG1655, and *atf1* gene from *R. opacus* PD630	Plasmid pTACHis18 (Km^R^; expression vector containing inducible promoter *P*_*tac*_, derived from an *E. coli*-*Rhodococcus* shuttle vector); N-limited conditions	Xiong et al. (2016a)
*R. jostii* RHA1	Levoglucosan	TAGs	Heterologous expression of *lgk* gene from yeast *Lipomyces starkeyi* YZ-215	Plasmid pTACHis18 (Km^R^; expression vector containing inducible promoter *P*_*tac*_, derived from an *E. coli*-*Rhodococcus* shuttle vector); N-limited conditions	Xiong et al. (2016b)
*R. opacus* PD630	Glucose	FAs	Overexpression of autologous thioesterases (TEs)	Plasmids pJAM2/TEs (Amp^R^ and inducible *P*_*ace*_); N-limited conditions	Huang et al. (2016)
*R. opacus* PD630	Glucose	FFAs FAEEs LCHCs	Deletion of *fadD*, *fadE*, *alk*-*1* genes Heterologous expression of *LPD05381* gene coding for TAG lipase from *R. opacus* PD630 and *APZ15_19700* gene coding for lipase-specific foldase from *Burkholderia cepacia* Overexpression of *LPD05217* (the main *fadD* gene) Heterologous expression of *adhE*^*mut*^ gene from *E. coli* and *ws* gene from *Marinobacter hydrocarbonoclasticus* Heterologous expression of *acr* gene from *Clostridium kluyveri* Codon-optimized *aar* and *ade* genes from *Synechococcus elongatus*	pROP1 plasmids derived from pCH (Km^R^, p15A origin; *R. opacus-E. coli* shuttle vector), genes are cloned under inducible promoter *P*_*ace*_ ; deletion of *fad* and alkane-1 monoxygenase genes using pK19mobsacB system; overexpression of *fadD* gene by replacing its promotor with inducible *P*_*ace*_ and substituting its start codon (GTG) with ATG; utilization of artificial ribosome binding site (RBS) sequences upstream of *ws* gene; N variable concentrations	Kim et al. (2019)
*R. opacus* PD630	Kraft lignin	TAGs	Heterologous expression of *WP_003972284* gene coding for the laccase enzyme from *Streptomyces coelicolor* *tatA* and *tatC* genes, *fasI* operon and *atf2* gene from *R. opacus* PD630	*E. coli-Rhodococcus* shuttle vector pBSNC9031 (Thio^R^ and replication origin from pNC903); *E. coli*-*Rhodococcus* shuttle vector pT2 (Apra^R^)	Xie et al. (2019)
*R. opacus* PD630	Lignin and lignin-derived aromatics	*cis,cis*-Muconate	Deletion of the genes: *Pd630*_*LPD06567* coding for muconate cycloisomerase, *Pd630_LPD00178* coding for cathecol 2,3-dioxygenase, *Pd630_LPD05451* coding for protocatechuate 3,4-dioxygenase Heterologous expression of the genes *ADF61496* coding for protocatechuate decarboxylase from *Enterobacter cloacae* and *ADF63617* coding for flavin prenyl transferase	pK18mob-pheS* used for the deletion of the genes in a PD630 strain with mutant phenylalanyl-tRNA synthase gene (pheS*) Insertion of the optimized genes in nonsense locus of endogenous plasmid using the pheS*-based genome editing strategy	Cai et al. (2020)
*R. jostii* RHA1	Lignin	Pyridine-dicarboxylic acids	Deletion of *pcaHG* genes coding for protocatechuate 3,4-dioxygenase Heterologous expression of *ligAB* genes coding for protocatechuate 4,5-dioxygenase from *Sphingobium* SYK-6, *praA* gene coding for protocatechuate 2,3-dioxygenase from *Paenibacillus* sp. JJ-1b, *dyp2* gene coding for peroxidase from *Amycolatopsis* sp. 75iv2	Homologous recombination using the vector pK18*mobsacB* to insert *ligAB* or *praA* genes in place of *pcaHG* genes pTipQC2 expression plasmid	Spence et al. (2021)
*R. ruber* Chol-4	4-androstene-3,17-dione	Testosterone	Deletion of *kshB* gene in the triple *R. ruber* mutant ∆*kstD1*,2,3 Heterologous expression of codon-optimized *17β-hsd* gene from the fungus *Cochliobolus lunatus*	Construction of the inducible expression vector pNVNIT (includes the inducible P_nitA_, the inductor is ε-caprolactam)	Guevara et al. (2019)

^a^The focus was not specifically on TAG accumulation but on the improved growth on lignin-derived substrates and/or improved tolerance towards lignin-derived stressors that could inhibit the lipid accumulation

## Adaptive laboratory evolution

Regarding biosynthetic and bioconversion strategies, several adaptive laboratory evolution (hereafter: ALE) approaches were applied to generate a set of *R. opacus* PD630 strains featured by optimized growth and TAG production and accumulation from substrates derived from lignocellulosic biomass (Kurosawa et al. 2015a, b; Yoneda et al. 2016; Henson et al. 2018). ALE experiments consisted in the construction of mutant populations starting from *Rhodococcus* spp. strain cultures which were sequentially transferred into new cultures, at regular intervals, in the presence of specific growth conditions and/or stressors (i.e., selective pressure) (Fig. 1). This re-inoculation procedure carried out several times promotes the selection and propagation of beneficial mutations that sustain improved growth rate and/or higher resistance/tolerance capacities (i.e., improved fitness) (Dragosits and Mattanovich 2013; Sandberg et al. 2019).

In particular, to expand growth substrates spectrum, an ALE approach was applied to improve glycerol utilization in the engineered xylose-fermenting *R. opacus* strain MITXM-61, originating from *R. opacus* PD630 (Kurosawa et al. 2014, 2015b). In this work, sequential transfers of liquid cultures were performed by supplying at each passage 100 g/L of glycerol as sole carbon source. This procedure allowed the isolation of the strain MITGM-173, which showed improved growth performance in the presence of glycerol as compared to the parental strain. Strain MITGM-173 also exhibited the ability to simultaneously metabolize glucose, xylose, and glycerol to produce a large number of TAGs. TAG production resulted to be higher when the glycerol was added after 2 days of growth on glucose and xylose. Although glycerol assimilation mechanism needs further investigation, the authors hypothesized that the improved lipid production could be due to the function of glycerol as direct precursor in TAG biosynthesis (Kurosawa et al. 2015b). In another work by the same authors, ALE experiments were performed to generate a *Rhodococcus* strain with enhanced tolerance to the inhibitors derived from lignocellulose hydrolysis, such as furans and phenols (Kurosawa et al. 2015a). Specifically, a *R. opacus* MITXM-61 evolved strain was obtained that was tolerant to lignin, 4-HB, and syringaldehyde. The mutant also showed improved growth performance and a higher amount of TAG production on lignocellulosic hydrolysates as compared to the parental strain.

More recent studies combined ALE with multi-omics approaches to identify novel targets for engineering *R. opacus* strains toward lignin valorization. Specifically, in two different papers, ALE was successfully applied to PD630 obtaining evolved strains with improved aromatic utilization and tolerance and lipid production (Yoneda et al. 2016; Henson et al. 2018). Comparative transcriptomics studies between the WT and the adaptively evolved strains showed that both strains upregulate gene clusters involved in the transformation of lignin-derived aromatic compounds to either catechol (CAT) or protocatechuate (PCA) which are subsequently converted through the β-ketoadipate pathway in succinyl-CoA and acetyl-CoA. Acetyl-CoA is the main precursor of lipid biosynthesis. Interestingly, in all adaptively evolved strains, the 36% of non-synonymous SNPs were identified in genes involved in redox reactions, suggesting that the redox state of the cell could be important for the improved tolerance and utilization of aromatic compounds. In particular, all mutant strains shared a non-synonymous SNP in the gene encoding for the superoxide dismutase (SOD), causing a 56% activity loss under in-vitro conditions (Henson et al. 2018). A lower SOD activity could increase the intracellular levels of superoxide radicals that participate in the oxidation of aromatic compounds catalyzed by mono- and dioxygenases (Gatti et al. 1994; Bugg 2001; Henson et al. 2018). An additional metabolomic study elucidated the biodegradation pathway of a lignin-derived aromatic compound, i.e., phenol, via quantitative flux balance analysis (FBA). In the presence of phenol, PD630 metabolic network showed strong metabolic fluxes through the TCA cycle, making this strain an ideal host for the production of metabolic intermediates such as acetyl-CoA and α-ketoglutarate for TAG production from lignocellulosic biomass (Roell et al. 2019).

## Heterologous gene expression

The production of specific proteins and functions in *Rhodococcus* spp. strains was achieved by developing several types of expression vectors based on cryptic (i.e., with unknown function) plasmids and transposable DNA elements tools for the insertion of cloned genes into chromosomes (Mitani et al. 2006). In the field of bioconversions, *Rhodococcus* strains were engineered to improve the utilization of cellulose and hemicellulose degradation products such as cellobiose, xylose, l-arabinose, and levoglucosan (Fig. 2A). The latter is an anhydrous sugar deriving from the pyrolysis of cellulose, and only few microorganisms are known to be able to metabolize it. With regard to the recombinant strains, *R. opacus* PD630 engineered with the *bglABC* operon from *Thermobifida fusca*, which encodes for two ABC sugar transport proteins (BglA and BglB) and a β-glucosidase (BglC). As a result, the recombinant strain exhibited both increased growth on cellobiose and improved lipid accumulation (up to 39% of the cell dry mass) as compared to the wild-type strain (Hetzler and Steinbüchel 2013).

Heterologous *xylA* and *xylB* genes, coding for d-xylose isomerase and xylulokinase respectively, from *Streptomyces padanus* MITKK-103 (Kurosawa et al. 2013) and from *Streptomyces lividans* TK23 (Xiong et al. 2012) conferred to *R. opacus* PD630 and *R. jostii* RHA1 the ability to produce lipids in presence of xylose as sole carbon source. The two works used different approaches to achieve the heterologous expression of *xyl* genes. Kurosawa et al. (2013) created a genomic library of *S. padanus* in *R. opacus* PD630 cells that were further screened for their ability to grow on xylose. Within this library, the strain Xsp8 showed the highest level of TAG accumulation. Characterization of the Xsp8 plasmid confirmed the presence of *S. padanus* genes homologous to *xylA* and *xylB*. The recombinant strain was able to grow simultaneously on glucose and xylose with final TAG accumulation corresponding to 45.8% of the cell dry mass (CDM). On the other hand, Xiong et al. (2012) cloned *xylA* and *xylB* genes from *S. lividans* inside the *E. coli-Rhodococcus* shuttle vector pNV18 downstream of the inducible *tac* promoter (P_*tac*_). Under nitrogen-limited condition, the lipid accumulation on xylose resulted to be up to 68.3% and 52.5% of CDM in PD630 and RHA1 recombinant cells, respectively (Xiong et al. 2012). These *Rhodococcus* strains were also engineered to obtain recombinant cells able to use l-arabinose for the growth. The genes *araB*, *araD*, and *araA* from *Streptomyces cattleya* NRRL 8057 (Kurosawa et al. 2015c) and from *Escherichia coli* K12 MG1655 (Xiong et al. 2016a) were expressed in *R. opacus* PD630 and *R. jostii* RHA1, respectively. The three genes code for the enzymes l-ribulokinase, l-ribulose-5-phosphate 4-epimerase, and l-arabinose isomerase which catalyze the reactions leading to l-arabinose transformation into d-xylulose-5-phosphate that can enter the pentose phosphate pathway. The engineered PD630 strain MITAE-348 exhibited good growth performance in the presence of high concentrations of l-arabinose (up to 100 g/L) and subsequent lipid accumulation (39.7% of the CDM). In the presence of a mixture of l-arabinose and d-glucose (1:1), the strain metabolized both sugars simultaneously with higher TAG production as compared to growth on the only l-arabinose, reaching a TAG content that corresponds to 42.0% of the CDM (Kurosawa et al. 2015c). In addition to the capacity to metabolize l-arabinose, recombinant cells of *R. jostii* RHA1 showed an increase in biomass production when the arabinose transporter operon *araFGH* was expressed, while higher lipid content (56.8% of the CDM) was obtained by expressing *atf1* gene, a key gene for TAG biosynthesis, from *R. opacus* PD630 (Xiong et al. 2016a). The heterologous expression approach was also successfully applied to *R. jostii* RHA1, conferring the capacity to metabolize the sugar levoglucosan by expressing the gene levoglucosan kinase (*lgk*) from the yeast *Lipomyces starkeyi* YZ-215. The gene *lgk* encodes a specific levoglucosan kinase that converts levoglucosan into glucose-6-phosphate, which enters the glycolysis pathway. As a result, the recombinant RHA1 cells acquired the capacity to grow on levoglucosan as sole carbon source. Despite the lipid accumulation rate on this substrate was lower, the final lipid content on levoglucosan resulted to be similar to that obtained on glucose, up to 43.54% of the cell dry mass (Xiong et al. 2016b).

## Overexpression of autologous genes

Overexpression of native genes in *Rhodococcus* spp. strains associated with the improvement of biosynthetic abilities was mainly performed by cloning these genes in episomal expression systems under the control of inducible promoters (e.g., P_tipA_ and P_ace_ inducible by thiostrepton and acetamide, respectively). The fatty acid biosynthesis was enhanced in *R. opacus* PD630 grown on glucose as sole carbon source by overexpression of autologous thioesterases (Huang et al. 2016). Thioesterases (TEs) are hydrolytic enzymes that break the thioester bond between acyl-ACP and fatty acid chain stopping the fatty acid elongation cycle. Released fatty acids can be converted to TAGs and other storage lipids. The authors found 4 putative TE genes in *R. opacus* PD630. Overexpression of TE2 and TE4 led to an increased lipid content reaching 46% and 44% of cell dry mass (CDM) respectively, whereas the control strain and the recombinant cells overexpressing TE1 and TE3 only reached 37% of CDM. Overexpression and deletion of additional genes involved in TAG metabolism, such as *atf1* and *atf2*, *pap2*, *tadD*, *ltp1*, and *nlpR*, and genes encoding a NADP^+^-dependent malic enzyme were conducted in order to clarify their functional role in the lipid metabolism in *R. opacus* PD630 and *R. jostii* RHA1 (Hernández et al. 2013, 2015, 2017, 2019; Mandal et al. 2019; MacEachran and Sinskey 2013). Although these works were not focused on the evaluation of metabolic and bioconversion improvement, the overexpression or deletion of the aforementioned genes led to changes in TAG accumulation and yield, suggesting a possible application in genetic and metabolic engineering.

## Genome-scale metabolic models and metabolic engineering

The representation of biological systems complexity by mathematical models can be used to describe and predict cell behavior and metabolism and therefore useful for designing metabolic engineering strategies for biosynthetic pathway assessment and optimization. In this respect, genome-scale metabolic models (GEMs), i.e., mathematical representations of the stoichiometry of the biochemical networks, can be used to integrate in one single model information retrieved from physiological studies, gene-protein-reaction association, metabolic flux analysis, and thermodynamic analysis of pathways (O’Brien et al. 2015). For instance, GEM can be used to redesign portions of the metabolic network and predict the production rate of a metabolite under specific operating conditions. Redesign the metabolic network can be achieved by imposing metabolic constraints and in silico by removing a given reaction from the GEM (e.g., gene knock-out mutant) or imposing uptake null for a nutrient in the growth media. In this regard, the GEM model iMT1174 was developed in *R. jostii* RHA1 and used to predict the accumulation rate of three types of carbon storage compounds (i.e., glycogen, polyhydroxyalkanoates, and triacylglycerols) using different carbon sources (glucose or acetate) and under growth conditions typically occurring in activated sludge bioreactor systems for wastewater recovery (Tajparast and Frigon 2015, 2018). Starting from the iMT1174 metabolic network, different objective functions were implemented based on a set of metabolic constraints such as minimization and maximization of the specific metabolic fluxes and minimization and maximization of ATP production rate and reducing redox potential (NADH). The predicted accumulation rate of storage compounds for each of the objective functions was further validated using ^13^C-metabolic flux analysis (^13^C-MFA) (Tajparast and Frigon 2015). These studies, taken together, represent the first effort of simulating and predicting *Rhodococcus* metabolic fluxes leading to the production and accumulation of valuable compounds with industrial interest.

A few metabolic engineering approaches were focused on the genetic and metabolic modification of *R. opacus* PD630 and *R. jostii* RHA1 to promote lignin bioconversion and enhance the production of valuable compounds. These works focused on the optimization of the bioconversion of lignin into TAGs (Xie et al. 2019), *cis,cis*-muconate (CCMA) (Cai et al. 2020), or pyridine-dicarboxylic acids (Spence et al. 2021) and are summarized in Fig. 2A. In particular, the production of TAGs from lignin in *R. opacus* PD630 was improved by optimizing (at both transcriptional and translation level) the heterologous expression of a laccase gene (from *Streptomyces coelicolor*) and the secretion system (i.e., Tat transporter components, TatA and TatC) that is needed for the laccase extracellular ligninolytic activity (Xie et al. 2019). Furthermore, to allocate more carbon into lipid biosynthesis, the type I fatty acid synthase (*fasI*) operon encoding the main enzyme involved in fatty acid biosynthesis in *Rhodococcus* (Schweizer and Hofmann 2004) and the diacylglycerol acyltransferase gene *atf2*, which catalyzes the final step of TAG production (Hernández et al. 2013) were overexpressed in the laccase-producing PD630 strain. As a result, the final recombinant strain showed tenfold increased growth on 1% w/v of insoluble kraft lignin as compared to the wild-type strain and exhibited a significantly enhanced lipid production (Xie et al. 2019). With regard to the bioconversion of lignin into *cis,cis*-muconate (CCMA), Cai et al. (2020) genetically modified *R. opacus* PD630 by introducing two genes from *Enterobacter cloacae*, which encode a protocatechuate decarboxylase and a phenyltransferase. The introduction of these enzymes was aimed at funneling lignin degradation intermediates (i.e., protocatechuate) to the catechol degradation pathway that leads to the production of CCMA through β-ketoadipate pathway. Genes involved in further CCMA degradation and in alternative degradation pathways of catechol and protocatechuate were deleted to optimize the CCMA accumulation from lignin and lignin-derived aromatics. Both deletion and insertion were obtained by applying a markerless gene deletion/insertion system on a *R. opacus* PD630 mutated in a phenylalanyl-tRNA synthase gene that is used as negative counter-selection marker.

Recently, lignin was also found to be converted in pyridine-dicarboxylic acids (PDCAs) by an engineered *R. jostii* RHA1, for possible new bioplastics development (Spence et al. 2021). In this work, a metabolic engineering approach was applied to RHA1 to re-route the protocatechuate (derived from lignin and lignin-derived compounds) into the production of PDCA. In particular, *ligAB* genes from *Sphingobium* SYK-6 or *praA* gene from *Paenibacillus* sp. JJ-1b were inserted in the place of pcaHG genes in the RHA1 chromosome by homologous recombination. This method simultaneously allowed to block the competitive β-ketoadipate pathway by deleting the gene coding for the first enzyme of the pathway (*pcaHG*) and to express the enzymes responsible for the production of pyridine-2,4-dicarboxylic acid (2,4-PDCA) and pyridine-2,5-dicarboxylic acid (2,5-PDCA), respectively. The additional heterologous expression of *dyp2* gene from *Amycolatopsis* sp. 75iv2, coding for a peroxidase in the mutant strain, significantly increased the rate of lignin oxidation and accumulation of 2,4-PDCA in the final recombinant RHA1 strain utilizing different types of lignin (Spence et al. 2021).

While these works were mainly focused on valuable compound production from lignin bioconversion, a study by Kim et al. (2019) described the optimization of *Rhodococcus* biosynthetic abilities with regard to the high value-added products (i.e., free fatty acids) deriving from TAGs biotransformation (Fig. 2A). With this aim, the genome scale metabolic modeling was combined with a systematic metabolic engineering strategy in order to predict *R. opacus* PD630 metabolism and to estimate the theoretical yields of fatty acids and their derivatives. In particular, the cultivation conditions of *R. opacus* were optimized to maximize the accumulation of TAGs from glucose metabolism and, after this, the metabolism of the strain was systematically analyzed and redesigned to enable higher production yield of fatty acids and biodiesel (i.e., fatty acid ethyl esters and long-chain hydrocarbons). The authors performed the deletion of specific genes to block intermediate or products degradative pathways, replacement of some native promoters to enhance the lipid production, and insertion of additional genes to introduce heterologous metabolic pathways involved in fatty acid ethyl ester accumulation. As a result, three recombinant strains were obtained which showed the highest ability ever described through fermentative processes to produce free fatty acids (FFAs, up to 50.2 g/L), fatty acid ethyl esters (FAEEs, up to 21.3 g/L), and long-chain hydrocarbons (LCHCs up to 5.2 g/L) (Kim et al. 2019).

An additional study by Guevara et al. (2019) described the genetic modification of *Rhodococcus ruber* Chol-4 to produce and accumulate testosterone from 4-androsterone-3,17-dione (AD) bioconversion (Fig. 2B). *Rhodococcus ruber* Chol-4 is well known as a steroid degrader, although it is not a model strain and therefore not extensively characterized in the literature. For this reason, unlike *Rhodococcus* strains PD630 and RHA1 for which molecular tools for genetic manipulation are well known and efficient, a novel method for gene deletion (based on pK18-derived plasmid) was developed to manipulate Chol-4 strain. A specific expression vector was also constructed for this strain by modifying the *Nocardia*-*E. coli* shuttle vector pNV119 harboring the inducible promoter P_nitA_. Using these methods, a Chol-4 strain deletion mutant was developed to prevent AD and testosterone degradation by blocking the steroid catabolic pathway. Then, the gene coding for the enzyme 17-ketosteroid reductase (17ß-HSD) from the fungus *Cochliobolus lunatus* was inserted to allow the biotransformation of AD into testosterone. The recombinant strain showed a molar conversion rate of AD to testosterone after 24 h of 48.2%, while no production was detected in the wild-type strain. When glucose was added to the growth medium as co-substrate to stimulate the nicotinamide cofactor regeneration, which is involved in the reaction, the molar conversion rate of AD to testosterone after 24 h increased to the 61.5% (Guevara et al. 2019).

## First synthetic biology approaches applied to *Rhodococcus* spp. strains

Recent advances in genomics and genome editing opened new perspectives for the engineering of *Rhodococcus* spp. strains based on genomic-scale rational design and synthetic biology approaches. In this context, specific genetic tools for tunable gene expression were characterized and developed to expand the ability to control and characterize gene expression in *Rhodococcus* spp. strains. Indeed, the construction of synthetic biology circuits and pathways relies on well-defined libraries of promoter components (i.e., ribosome-binding site and regulatory regions) that in a controlled and predictable way can drive the expression of target genes. Promoter mini-pools with different activity levels were developed in *R. opacus* PD630 and *R. ruber* TH by using different fluorescent reporter genes, β-galactosidase (LacZ), and nitrilase (NHase) as different promoter activity probes (Jiao et al. 2018; DeLorenzo et al. 2017). In detail, the three chemically inducible promoters pBAD, pTet, and pAcet deriving from *E. coli* or *Mycobacterium smegmatis* were optimized for *R. opacus* PD630 as well as two classes of metabolite sensors responsive to nitrogen levels and specific aromatic monomers, typically found in depolymerized lignin, e.g., phenol, 4-hydroxybenzoic acid, guaiacol.

Although promoter libraries, reporter genes, and shuttle vectors have been developed for *Rhodococcus* as genetic tools, the genome editing of these bacteria has been hampered by its high GC content (~ 70%) and by low transformation/recombination efficiency (DeLorenzo et al. 2017, 2018; Jiao et al. 2018; Liang et al. 2020). Recently, DeLorenzo et al. (2018) developed a recombineering method for site-specific gene insertion and deletion, which was based on the activity of the bacteriophage recombinases Che9c60 and Che9c61 in PD630. This study provided the groundwork to develop a CRISPR/Cas9-mediated triple-plasmid recombineering system for genetic engineering of *R. ruber* TH. Specifically, the stable mutant strain *R. ruber* THY was obtained by using this CRISPR/Cas9-based method which featured an increased acrylamide production capacity as a result of the nitrile hydratase point mutation and a by-product gene deletion. Unlike *R. opacus* strains, the by-pass of restriction-modification system seemed to be necessary for efficient transformation of the *R. ruber* strains (Liang et al. 2020). Additional CRISPR-based approaches concerned the utilization of a CRISPR interference (CRISPRi) and a codon-optimized version of the catalytically dead Cas9 (dCas9) deriving from *Streptococcus thermophilus* (dCas9_Sth1_), as a system for gene expression control in *R. opacus* PD630. Like in *Mycobacterium tuberculosis*, the repression ability of dCas9_Sth1_ was independent of the distance of the sgRNA (single-guide RNA) from the transcriptional start site (DeLorenzo et al. 2018). In a very recent study, the possibility to utilize genetic parts to build genetic circuits to perform “logic function” in *R. opacus* was also explored. Genetic logic circuits—AND, NAND, and IMPLY—were constructed in PD630 strain combining a T7 RNA polymerase-based expression system (T7 RNAP), three novel synthetic IPTG-dependent promoters, and four aromatic sensors (DeLorenzo and Moon 2019). Apparently, while these recent studies provide the groundwork of the application of synthetic biology strategies to *R*. *opacus* PD630, further work is required to genetically characterize other *Rhodococcus* species which are known to be highly diversified in terms of genomic contents, metabolic pathways, and evolutionary adaptations and therefore possibly usable for different biotechnological applications (Cappelletti et al. 2019).

## Conclusions

Members of *Rhodococcus* genus are able to use low-cost and renewable resources as bioconversion substrates for the production of high value-added compounds. In this field, *Rhodococcus* bioconversion of lignocellulosic biomass into neutral lipids, i.e., triacylglycerols (TAGs) for biofuel generation, represents the most prominent example. Genetic manipulation strategies based on approaches of adaptive laboratory evolution (ALE) and expression systems of specific autologous or heterologous genes have successfully led to the generation of *Rhodococcus* spp. strains with improved biosynthetic activities in terms of production yields, types and number of metabolized substrates, tolerance/resistance to lignin-derivative stressors (Table 1). The mutagenesis experiments were in some cases combined with multi-omic approaches and genome-based metabolic modeling to provide integrative and system-level information about metabolic pathways involved in the biosynthetic process in *R. jostii* RHA1 and *R. opacus* PD630 and genetic traits to be targeted in possible genome editing approaches. Interestingly, a few metabolic engineering approaches were successfully applied to *R. opacus* PD630 strains (by deleting genes that catabolize the desired product or metabolic intermediate and by introducing new functions), which led to the development of strains characterized by outstanding capacities, i.e., the highest efficiency in producing fatty acids and related products ever reported. Recent breakthroughs in genetic engineering of *Rhodococcus* have also included the use of synthetic biology platforms and new approaches for genome editing (CRISPR/Cas9 and recombineering) mostly targeting *R. opacus* strain PD630. Despite that more efforts are needed to expand system and synthetic biology tools for genome-scale engineering of *Rhodococcus* species different from the model ones, the application of first metabolic engineering strategies and novel molecular toolkits has highlighted the great potential of engineered *Rhodococcus* spp. strains in biotechnological and industrial applications.
